# Development of reverse-transcription loop-mediated isothermal amplification assay for rapid detection of novel avian influenza A (H7N9) virus

**DOI:** 10.1186/s12866-014-0271-x

**Published:** 2014-11-14

**Authors:** Juan Liu, Qing-Gong Nian, Jing Li, Yi Hu, Xiao-Feng Li, Yu Zhang, Yong-Qiang Deng, Shun-Ya Zhu, Qing-Yu Zhu, E-De Qin, Tao Jiang, Cheng-Feng Qin

**Affiliations:** Department of Virology, State Key Laboratory of Pathogen and Biosecurity, Beijing Institute of Microbiology and Epidemiology, No.20 Dongda Street, Beijing, Fengtai District 100071 China; Department of Transfusion Medicine, PLA Air Force General Hospital, Beijing, 100142 China

**Keywords:** Influenza virus, H7N9 subtype, Reverse transcription-loop-mediated isothermal amplification, Molecular diagnosis

## Abstract

**Background:**

The emerged human infection with avian influenza A (H7N9) virus in China since 2013 has aroused global concerns. There is great demand for simple and rapid diagnostic method for early detection of H7N9 to provide timely treatment and disease control. The aim of the current study was to develop a rapid, accurate and feasible reverse-transcription loop-mediated isothermal amplification (RT-LAMP) assay for detection of H7N9 virus.

**Results:**

The detection limits of the H7- and N9-specific RT-LAMP assay were both approximately 0.2 PFU per reaction. No cross-reactivity was observed with other subtype of influenza viruses or common respiratory viral pathogens. The assay worked well with clinical specimens from patients and chickens, and exhibited high specificity and sensitivity.

**Conclusions:**

The H7/N9 specific RT-LAMP assay was sensitive and accurate, which could be a useful alternative in clinical diagnostics of influenza A (H7N9) virus, especially in the hospitals and laboratories without sophisticated diagnostic systems.

## Background

Since February 2013,a novel avian influenza A (H7N9) virus has emerged in China, resulting in human infections [1]. As of 7 Sept 2014, the Chinese CDC reported 452 laboratory-confirmed human cases [2]. According to the etiology and gene traceable results, H7N9 avian flu virus is a new recombinant virus [3–5]. Human infection with avian influenza A (H7N9) virus usually results in an influenza-like illness (ILI) with symptoms such as fever, cough with little to no sputum production, accompanied by headache, muscular soreness, and general malaise [6]. Most patients presented with rapidly progressing severe lower respiratory tract infections. Considering that the novel avian H7N9 is characteristic of mammalian adapted, scientific community widespreadly concerns that the emerging reassorted virus could cause a new influenza pandemic [7,8].

Several newly published researches indicated that the H7N9 virus exhibited human-type receptor-binding ability and could replicate in mammals [9–11]. H7N9 virus could invade epithelial cells in human lower respiratory tract and pneumonocytes [11]. These biological characterizations of H7N9 virus increase the pandemic risk especially that the virus acquires the ability of transmitting readily among humans, and the lack of pre-existing immunity to virus of this subtype among humans [12]. Therefore, the risk of a pandemic caused by avian H7N9 virus requires rapid detection methods.

Currently, the available detection methods for H7N9 virus include virus isolation and real-time RT-PCR assay [6]. The serological detection and real-time PCR were recommended to detect avian influenza A (H7N9) in 2013 by World Health Organizatin (WHO) [13]. Considering the requirement for specific equipment and trained operators of real-time RT-PCR assay, there is great demand for simple, rapid and sensitive diagnosis method for early detection of H7N9 to provide timely treatment and disease control. The loop-mediated isothermal amplification (LAMP) method, first described in 2000, has been proved to be a rapid, accurate, and cost-effective diagnostic method for infectious diseases [14–16]. Previously, the LAMP methods have been applied for detection of different subtypes of influenza viruses, including avian H3, H5, H7, H9, the 2009 H1N1 influenza virus, seasons type A or B influenza virus, and H3 swine influenza virus [17–23]. All these assays showed high specificity, efficiency, and sensitivity that were similar to or even higher than real-time PCR assays. These studies exhibited the potential of LAMP as a routine diagnostic method for influenza infections.

In this study, a H7N9-specific RT-LAMP assay targeting the HA or NA gene of avian influenza A (H7N9) virus was developed and evaluated with clinical throat swab and avian samples. The results demonstrated that the RT-LAMP assay was sensitive and accurate, which could be a useful alternative in clinical diagnostics of influenza A (H7N9) virus, especially in the hospitals and laboratories without sophisticated diagnostic systems.

## Results

The primer sets of H7 or N9 specific RT-LAMP comprise two outer primers (F3 and B3), two inner primers (FIP and BIP), and one loop primer LF that recognize seven distinct regions on the target sequence of HA or NA gene. Considering that the genomic stability of the emerging reassorted H7N9 was still unclear, the target sequences of the primers were optimized to avoid covering the hot spot in HA or NA gene, such as receptor binding domain or membrane fusion loop in HA gene. After evaluation with different dilutions of H7N9 viral RNA, the primer set (Table 1) was selected.

**Table 1:** **RT-LAMP primer sets designed for detection of H7N9 virus**

**Primer**	**Position** ^**a**^	**Length**	**Sequence (5′-3′)**
H7F3	1331-1350	20	AGCATACAATTGATCTGGCT
H7B3	1509-1529	21	ATTCTATTTTGCATTGCCTCT
H7FIP^b^	(1398-1419) + (1356-1375)	44	GCCATCTTCTTCAGCATTCTCTCT*TTTT*AGAAATGGACAAACTGTACG
H7BIP	(1443-1466) + (1489-1508)	44	CAAGTGTGATGATGACTGTATGGC*TTTT*TTCCCTGTATTTGCTGTGATC
H7LF	1376-1395	20	CAGCTGTCTTTTCACTCGTT
N9F3	1054-1071	18	GATGGGGCTAACACTTGG
N9B3	1228-1245	18	ATAGCAGTCCCCTTCAGC
N9FIP	(1115-1138) + (1076-1091)	44	TCAATGCATTTGGCACTTTTAACAT*TTTT*TAGGGAGGACAATAAGCAC
N9BIP	(1146-1168) + (1201-1221)	44	TAGATCAAAGCCCATTCAAGGTC*TTTT*GTCCATGAAAGATCCACTGTA
N9LF	1092-1113	22	CTCGTATCCAGACCTCGAGGCT

^a^H7N9 strain A/Anhui/1/2013 [GISAID: EPI439507 & EPI439509].

^b^FIP and BIP primer are long primers containing two separate recognition sequences with a TTTT linker (italics).

**Figure 1:**
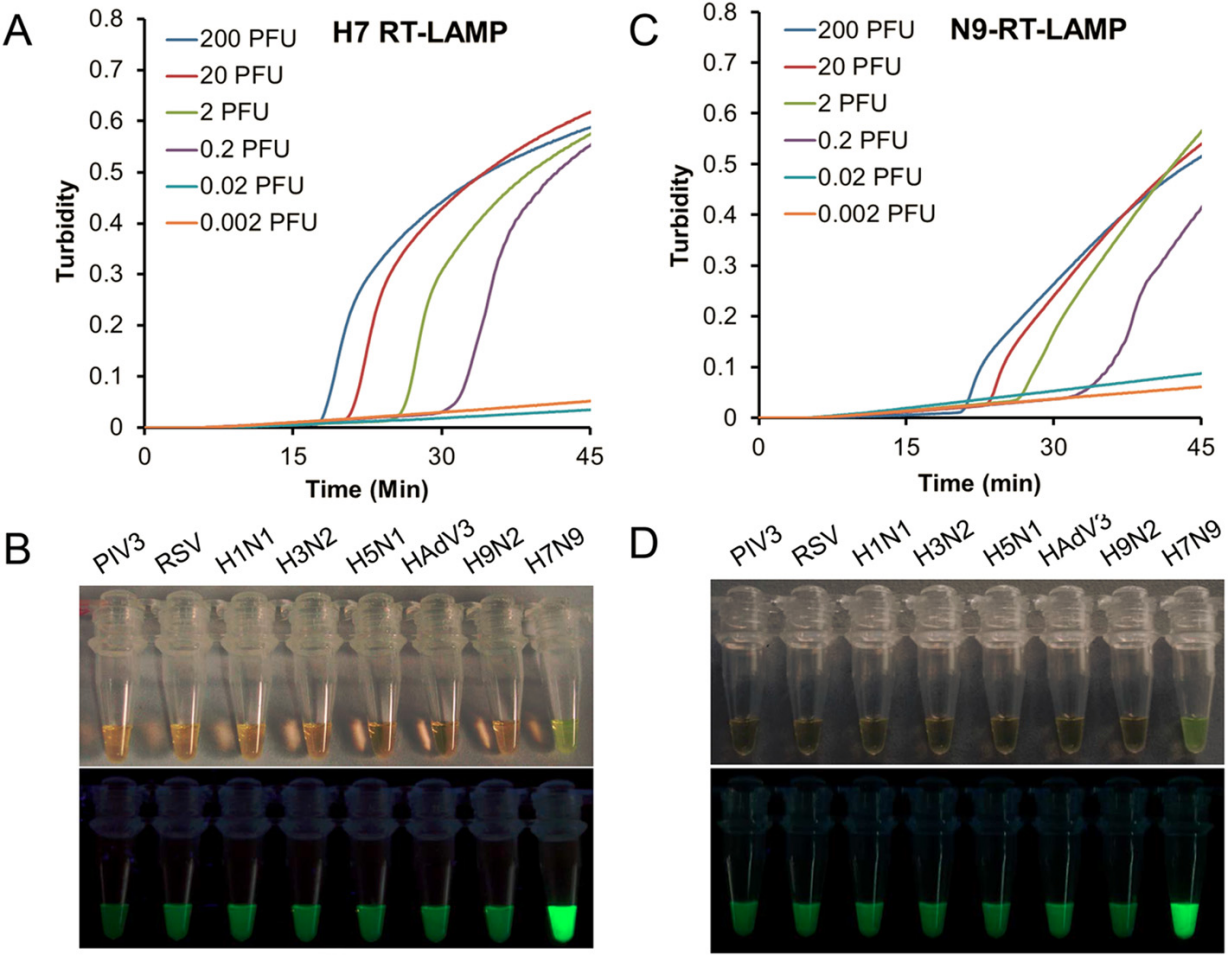
**Sensitivity and specificity of the H7/N9 -specific RT-LAMP assay.** Amplification curves of the H7/N9-special RT-LAMP were performed with 10-fold serial dilutions of viral RNA (from 200 to 0.002 PFU per reaction). Specificities of H7 and N9 RT-LAMP were tested by using direct visual detection of RT-LAMP with RNA extracted from viral culture of H1N1, H3N2, H5N1, PIV3, H9N2, RSV, HAdV-3 and H7N9. **(A)** Detection limit of H7-specific RT-LAMP was 0.2 PFU per reaction. **(B)** The color changes under natural or UV light were only observed in the tube of H7N9 viral RNA by using H7-specific RT-LAMP assay. **(C)** Detection Limit of N9-specific RT-LAMP was 0.2 PFU per reaction. **(D)** The color changes were observed for N9-specific RT-LAMP reaction with H7N9 viral RNA. No color change was seen in RT-LAMP with other subtype of influenza viral RNA and other three respiratory viruses under natural or UV light.

The detection limits of the H7 and N9 specific RT-LAMP assay were both 0.2 PFU per reaction (Figure 1A and C), which was 10-fold-higher sensitive than that of real-time RT-PCR assay recommended by WHO (Table 2). Furthermore, the cross-reactivity tests with seven respiratory viruses also revealed the high specificity of the H7/N9 RT-LAMP assay.

**Table 2:** **Comparison of detection of H7N9 virus by using RT-LAMP and rRT-PCR assay**

	**Strain**	**Virus Titer (PFU/reaction)**	**rRT-PCR(H7)(Ct)**	**rRT-PCR (N9) (Ct)**	**RT-LAMP (H7) (Tp,min)**	**RT-LAMP (N9) (Tp, min)**
H7N9	A/Anhui/1/2013	200	26.11	25.89	17.9	21.1
		20	29.35	29.71	20.4	23.5
		2	33.24	32.16	25	27.6
		0.2	ND^a^	>35	28.9	37.8
		0.02	ND	ND	ND	ND
		0.002	ND	ND	ND	ND

^a^ND, not detectable.

## Discussion

The RT-LAMP assay could finish within 45 minutes. By using fluorescent detection reagent, the positive amplification could be observed by color change. As shown in the Figure 1B and D, the color of positive reaction (H7N9) change from orange to green under natural light, while the positive reaction also produced bright green fluorescence under UV light. For negative sample such as H1N1, H3N2, H5N1, PIV3, H9N2, RSV and HAdV-3 virus, the color of reaction mixture did not change either under normal light or under UV light.

The H7/N9-specific RT-LAMP assay was further evaluated using clinical samples and chicken samples compared with rRT-PCR assay. The RT-LAMP results showed that 17 samples were tested to be positive and the other 10 samples were negative for H7N9, respectively, which could be confirmed by rRT-PCR assay. No false positive and false negative were observed. By using these 27 samples, 100% diagnostic sensitivities were achieved with H7 or N9 specific RT-LAMP assay compared with rRT-PCR assays, which showed that RT-LAMP results were consistent with that of rRT-PCR. The LAMP method aimed to detect 2013 avian influenza H7N9 showed reliable results in the detection of avian and human clinical samples.

Previously, two RT-LAMP systems had been developed for detection of avian influenza subtype H7 virus [17,23]. Currently, some other RT-LAMP assays for detection of H7N9 virus were also developed [24–28]. These assays showed excellent sensitivities and specificities. Our H7/N9-specific RT-LAMP assays, as well as those published RT-LAMP methods, were very useful for the early detection of suspected H7N9 infection, and could contribute to the prevention and control of H7N9 epidemics and potential pandemic in China and other asian countries.

## Conclusions

The current study described a rapid, accurate and feasible H7/N9 specific RT-LAMP assay with perfect sensitivities and high specificities. It was simple and easy to perform. This assay have the potential to provide useful tools for detection of novel H7N9 virus especially in resource-limited setting, which will play important roles in the prevention and control of spread of novel H7N9 infection.

## Methods

### Samples

A total of 5 clinical throat-swab samples were collected from suspected patients with H7N9 infection. All samples were collected between 2 days and 7 days after the onset of illness. The clinical samples used in this study were appropriately anonymized. All individuals participating in the study gave written informed consent. Local ethical approval was obtained and guidelines were followed for the use of clinical material and accession to diagnostic results. A total of 22 mixed chicken stool samples were collected randomly from live poultry market in Nanjing, Jiangsu and got permission by the chicken farmer and administrator of poultry market. The stool samples were added by phosphate-buffered saline (PBS; 0.02 mol/L; pH 7.2) to make a 10% stool suspension and then centrifuged at 13,000 g for 5 min. The suspension was subsequently processed for RNA extraction.

The collection of samples for the present study was approved by the Ethical Committee of the State Key Laboratory of Pathogen and Biosecurity (Beijing, China), and the experimental procedures were carried out in strict accordance with guidelines on animal ethics and welfare.

### Viruses

The H7N9 virus strain A/Anhui/1/2013, kindly provided by Chinese national influenza center (CNIC), was used as reference virus in the current study. Seven respiratory viruses, including human influenza virus H1N1 virus strain A/California/07/2009, H3N2 virus strain A/Beijing/073103/2009, H5N1 virus strain A/Vietnam/1194/2004, avian H9N2 virus strain A/chicken/Nanjing/1/2013, and other important respiratory viruses including type 3 parainfluenza virus (PIV3) strain BJ/01/2005, respiratory syncytial virus (RSV) strain BJ/01/2004 and human adenovirus type 3 (HAdV-3) strain GB, were used to evaluate the specificity of the assay.

### Primers design of RT-LAMP

The nucleotide sequences of complete genome of H7N9 strains were retrieved from the GenBank and GISAID (Global Initiative on Sharing All Influenza Data), and aligned by using the ClustalX multiple sequence alignment program. The RT-LAMP assay primers were designed on the basis of the H7N9 HA or NA alignments by using the LAMP primer designing software PrimerExplorer (http://primerxplorer.jp/e/). The feasibility of all sets of primers was then subsequently validated by BLAST program (http://blast.ncbi.nlm.nih.gov/Blast.cgi) and ClustalW software [29]. The primers were synthesized by Life Technologies (Shanghai) Ltd.

### RNA extraction

Total RNA was extracted from 200 μl of the clinical samples or viral culture supernatant by using the RNeasy® Mini Kit (Qiagen, Hilden, Germany) according to the instructions of the manufacturer. The RNA was eluted in a final volume of 50 μl of RNase-free water and stored at -80°C until used.

### RT-LAMP

The RT-LAMP reaction was carried out by using the Loopamp RNA amplification kit (Eiken Chemical, Tokyo, Japan). The reaction system contained 5 μl of total RNA, 40 pmol each of the primers FIP and BIP, 5 pmol each of the outer primers F3 and B3, 20 pmol of LF, 12.5 μl of 2 × Reaction Mix, 1 μl of Enzyme Mix and 1 μl of Fluorescent regent. The reaction mixture was incubated at 63°C for 45 min in a Loopamp real-time turbidimeter LA-320 (Eiken Chemical, Tokyo, Japan) and followed by heating at 80°C for 5 min to terminate the reaction. The optical density data of each reaction was real-time recorded every 6 s. The threshold of the turbidity for positive sample was defined at 0.1. The time of positivity (Tp) was determined when the turbidity value increased above the threshold.

### Real-time RT-PCR

As compared with the RT-LAMP detection, real-time RT-PCR (rRT-PCR) was carried out with the set of specific primers and probe for the detections of influenza A H7N9 virus recommended by WHO. The rRT-PCR was performed with the One-Step PrimeScript™ RT-PCR Kit (Takara, Dalian, China) in the LightCycler 2.0 system (Roche, Mannheim, Germany) in a 20 μl mixture containing 2 μl of total RNA, 10 μl of 2 × One Step RT-PCR Buffer III, 0.4 μl of Ex Taq, 0.4 μl of PrimeScript™ RT Enzyme Mix II, 0.8 μM of forward primer, 0.8 μM of reverse primer and 0.8 μM of probe. The reaction was performed for 5 min at 42°C, followed by 20 s at 95°C, with a subsequent 40 cycles of amplification (95°C for 5 s, 55°C for 20 s). Fluorescence was recorded at 55°C.

### Sensitivity and specificity of the assays

The sensitivities of the RT-LAMP assay and rRT-PCR assay were analyzed using 10-fold serially dilutions of viral RNA. The H7N9 virus strain A/Anhui/1/2013 was used to evaluate the sensitivity of RT-LAMP assay developed in the current study. The concentrations of viral RNA were serial diluted from 200 PFU to 0.002 PFU per reaction, respectively. The specificity of the assay was evaluated by cross-reactivity tests with seven respiratory viruses, including human influenza H1N1 virus, H3N2 virus, H5N1 virus, avian H9N2 virus, PIV3, RSV and HAdV-3.

### Evaluation with clinical samples

The H7/N9-specific RT-LAMP assay was evaluated using clinical samples from suspected patients with H7N9 infection and chicken stool samples collected from local live poultry markets. A total of 27 samples were tested for the presence of H7N9 virus by using the RT-LAMP assay and the real-time RT-PCR assay. Cp value of rRT-PCR less than 35 was judged as positive. Positive standard was identified as emergence of the positive curve or the color change within 45 minutes in the RT-LAMP assay.

## Abbreviations

RT-LAMP: Reverse-transcription loop-mediated isothermal amplification
PFU: Plaque Forming Unit
RT-PCR: Reverse transcription polymerase chain reaction
PIV: Parainfluenza virus
RSV: Respiratory syncytial virus
HAdV: Human adenovirus
Ct: Cycle threshold value
Tp: time of positivity
